# Safety and efficacy of acupuncture for anxiety and depression in patients with heart failure: A protocol for systematic review and meta-analysis

**DOI:** 10.1097/MD.0000000000031822

**Published:** 2022-11-18

**Authors:** Cheng Wang, Jia Wang, Rui Shi, Keying Yu, Miao Zhang, Ruozhu Lu, Mingpeng Shi, Yue Deng

**Affiliations:** a Changchun University of Chinese Medicine, Changchun, China; b Affiliated Hospital, Changchun University of Chinese Medicine, Changchun, China; c School of Traditional Chinese Medicine, Changchun University of Chinese Medicine, Changchun, China.

**Keywords:** acupuncture, anxiety, depression, heart failure, meta-analysis, protocol

## Abstract

In recent years, with the increase of patients with coronary heart disease, the number of patients with heart failure (HF) has also gradually increased. Coronary heart disease is one of the most common causes of HF. Anxiety and depression are frequent psychological disorders in patients with HF. Studies have shown that anxiety and depression can affect the quality of life of patients with HF, and can increase hospitalization and mortality. Conventional pharmacotherapy and psychotherapy have certain limitations. Acupuncture has therapeutic effects on heart disease, anxiety and depression, and has been widely used to relieve symptoms in patients with HF. This protocol aims to evaluate the safety and efficacy of acupuncture for anxiety and depression in patients with HF.

**Methods:**

We will search the following databases: PubMed, Web of Science, Springer Cochrane Library, EMBASE, MEDLINE, WHO international clinical trials registry platform, China National Knowledge Infrastructure database, Wan Fang database, Chinese scientific journal database and Chinese Biomedical Literature Database. The databases will be searched from initiate to October 1, 2022. Two reviewers will screen and document eligible studies based on inclusion and exclusion criteria. Two reviewers will independently perform data analysis and bias risk assessment. Review Manager version 5.4 software will be used for meta-analysis.

**Results:**

This study will explore the efficacy and safety of acupuncture for anxiety and depression in patients with HF.

**Conclusion:**

The results of this study will provide high-quality evidence for evaluating the safety and efficacy of acupuncture for anxiety and depression in patients with HF.

## 1. Introduction

Heart failure (HF) is an increase in intracardiac pressure or insufficient cardiac output due to abnormalities in the structure or function of the heart. The main symptoms of HF are breathlessness, ankle swelling and fatigue.^[1]^ Hypertension, obesity, coronary artery disease and other risk factors may lead to HF. Coronary heart disease is one of the most common causes of HF.^[1–3]^ The incidence of HF is mainly among the elderly. In recent years, with the growth of the aging population, the incidence of the disease has been increasing.^[1,4]^ Anxiety and depression are common psychological disorders in patients with HF. Studies have shown an association between anxiety, depression and HF.^[5,6]^ Long-term psychological disorders not only affect the quality of life of HF patients but also lead to increased hospital admissions and mortality.^[7,8]^ At the same time, the continuous progress of HF will also increase the degree of anxiety and depression in patients. Therefore, anxiety and depression in patients with HF should be treated and intervened in time.^[9]^ Clinically, HF, anxiety and depression are often treated independently. Modern medical treatments for anxiety and depression in HF patients are mainly antidepressants and cognitive behavioral therapy.^[10–12]^ Although these treatments have certain positive effects, there are also problems such as unstable efficacy or drug side effects.^[13]^

As a traditional therapy, acupuncture has been widely used to treat various heart diseases.^[14]^ There is evidence that acupuncture can enhance the cardiac function of patients with HF, and can improve the therapeutic effect and prognosis when combined with western medicine.^[15–17]^ In addition, some studies have shown that acupuncture is effective in treating anxiety and depression.^[18,19]^ However, the efficacy and safety of acupuncture for anxiety and depression in patients with HF remain unclear. It still needs further exploration. Therefore, we will conduct a systematic review and meta-analysis to provide a reference for clinicians.

## 2. Methods

### 2.1. Study registration

This systematic review protocol has been registered with the International Platform of Registered Systematic Review and Meta-Analysis Protocols (PROSPERO). The approved registration number is CRD42022365065. This protocol is based on the Preferred Reporting Items for Systematic Reviews and Meta-analysis Protocols (PRISMA-P) statement guidelines.^[20]^

### 2.2. Types of studies

We will collect published randomized controlled trials (RCTs) related to acupuncture for anxiety and depression in patients with HF, and there are no restrictions on language. These RCTs will be included, while non-RCTs, comments, Case reports, cohort studies, cross-sectional studies, animal experiments and reviews will be excluded.

### 2.3. Types of patients

All HF patients with anxiety and depression will be included in the study without any age, gender, or region restrictions.

### 2.4. Types of interventions

#### 2.4..1. Experimental interventions.

The patients in the experimental group will receive the intervention of acupuncture or acupuncture combined with other treatment methods. There are many forms of acupuncture treatment, such as manual acupuncture, warm needling, scalp acupuncture, electroacupuncture, fire needling, auricular acupuncture, or elongating needles.

#### 2.4..2. Control interventions.

The patients in the control group will receive non-acupuncture interventions, such as sham acupuncture, placebo, or other interventions. The acupoint numbers, retaining time, and frequency will not be restricted in this protocol.

### 2.5. Types of outcome measures

#### 2.5..1. Primary outcomes.

The primary outcome will be assessed by an anxiety or depression scale, such as the Hamilton Anxiety/Depression Scale.

#### 2.5..2. Secondary outcomes.

Secondary outcomes will include improved in patient symptoms and incidence of adverse events.

### 2.6. Data source and search strategy

We will search the following ten databases, including PubMed, web of science, Springer Cochrane Library, EMBASE, MEDLINE, WHO international clinical trials registry platform, China National Knowledge Infrastructure database, Wan Fang database, Chinese scientific journal database and Chinese Biomedical Literature Database. The databases will be searched from initiate to October 1, 2022, and the publishing language will be restricted to Chinese and English.

Content related to the subject words “acupuncture,” “heart failure,” “anxiety,” and “depression,” will be searched in the database. The search terms will be adapted appropriately to conform to the different syntax rules of the different databases. We will follow the guidelines from the Cochrane handbook. The Search strategy for PubMed is shown in Table 1, and similar strategies will be built and applied for other databases.

**Table 1:** The search strategy for Pub Med database.

Number	Search terms
#1	heart failure[Title/Abstract]
#2	congestive heart failure[Title/Abstract]
#3	HF[Title/Abstract]
#4	patients with heart failure[Title/Abstract]
#5	chronic heart failure[Title/Abstract]
#6	OR #1-#5
#7	acupuncture therapy[Title/Abstract]
#8	acupuncture[Title/Abstract]
#9	needling[Title/Abstract]
#10	electroacupuncture[Title/Abstract]
#11	fire needle[Title/Abstract]
#12	warm acupuncture[Title/Abstract]
#13	scalp acupuncture[Title/Abstract]
#14	fast acupuncture[Title/Abstract]
#15	skin acupuncture[Title/Abstract]
#16	bloodletting[Title/Abstract]
#17	catgut embedding[Title/Abstract]
#18	OR #7-#17
#19	anxiety[Title/Abstract]
#20	depression[Title/Abstract]
#21	depressive symptom[Title/Abstract]
#22	depressive disorder[Title/Abstract]
#23	OR #19-#22
#24	randomized controlled trial[Title/Abstract]
#25	trial[Title/Abstract]
#26	randomized[Title/Abstract]
#27	OR #24-#26
#28	#6 and #18 and #23 and #27

### 2.7. Data collection and analysis

#### 2.7..1. Study selection and data extraction.

**Figure 1.**
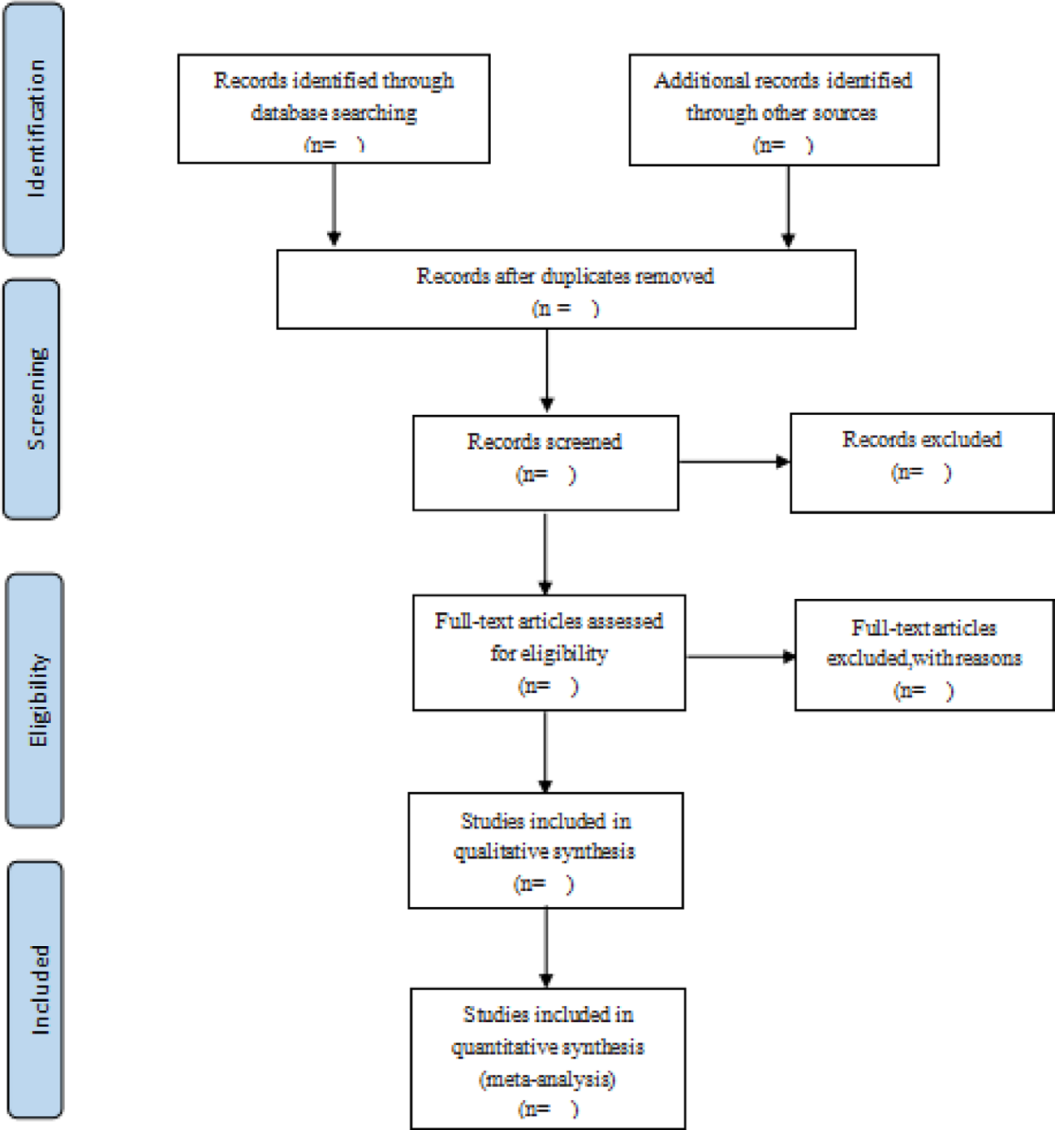
The PRISMA flow chart of the selection process. PRISMA = preferred reporting items for systematic reviews and meta-analysis.

Two researchers will independently screen the retrieved titles and abstracts according to exclusion and inclusion criteria and obtain the full text. Details of studies that meet the requirements will be recorded, and studies that do not meet the criteria will be excluded and the reasons recorded. If disagreements arise during this process, they will be thoroughly discussed and then arbitrated by a third investigator. The entire literature inclusion process will refer to the Cochrane Collaboration System Evaluator Manual (5.1.0). EndNote X9 will be used to manage the retrieved studies, and the relevant information will be recorded using an Excel spreadsheet. The screening flow diagrams of this study will be shown in Figure 1.

Two researchers will independently extract useful information from eligible studies and record them in a collection form after induction. The following information will be recorded: author’s name, time of publication, number of participants, country of study, information on participant characteristics (e.g., average age, gender, time of diagnosis, etc), relevant content of the intervention (e.g., method of acupuncture, depth, frequency, duration of treatment, etc), outcomes (e.g., degree of symptom improvement, changes in the scale, adverse event rate, etc). Disagreements arising will be resolved by a third researcher after discussion. If necessary, we will contact the authors of the paper.

#### 2.7..2. Risk of bias assessment.

Two reviewers will independently assess the risk of bias for each selected study using the Cochrane bias risk tool. They will be assessed in 7 areas: random sequence generation, allocation concealment, blinding of participants and personnel, incomplete outcome data, blinding of outcome assessment, selective reporting and other biases. All trials will be divided into 3 levels: low risk of bias, high risk of bias and unclear bias. In the event of any disagreement between the 2 reviewers on the risk assessment and after discussion, consensus cannot be reached, and the third reviewer will make the final decision.

#### 2.7..3. Management of missing data.

If study data and other information are missing, we will attempt to contact the study authors for relevant information. These studies were excluded when the information could not be supplemented.

#### 2.7..4. Data synthesis and assessment of heterogeneity.

RevMan V.5.4 software will be used for data analysis and synthesis. We will analyze dichotomous data using a Risk ratio with 95% CIs.For continuous data, a mean difference or a standard mean difference with 95% CIs will be applied for analysis. We will apply the ×2 test or the *I*^2^ test to assess heterogeneity. When there was significant heterogeneity between studies (*P* < .05, *I*^2^ ≥ 50%), we performed a random effects model. If heterogeneity is low, a fixed effects model will be used. When heterogeneity occurs, subgroup analysis or sensitivity analysis will be performed to assess the source of heterogeneity.

#### 2.7..5. Assessment of reporting biases.

If 10 or more studies are included, funnel plots and the Egger test will be used to assess reporting bias and then we will interpret this result cautiously.

#### 2.7..6. Subgroup analysis.

we will explore its source through subgroup analysis if there is significant heterogeneity. The following aspects will be considered: age, gender, modality of intervention and degree of depression. If the data is insufficient, a qualitative synthesis will be conducted.

#### 2.7..7. Sensitivity analysis.

If data are sufficient, we will perform a sensitivity analysis to test the robustness and reliability of the results, and certain low-quality or unblinded studies will be excluded.

#### 2.7..8. Assessment of quality of evidence.

We will use the Grading of Recommendations Assessment, Development, and Evaluation to assess the quality of evidence. The quality of evidence will be divided into 4 levels: high, moderate, low and very low.

## 3. Discussions

HF is a disease that seriously affects the quality of life of patients and brings a great financial burden to patients. Anxiety and depression are common complications in patients with HF and are associated with the development of HF. HF with anxiety and depression can make treatment more difficult. Psychotherapy and drug therapy are generally used to treat anxiety and depression in patients with HF. However, medication has certain side effects, and psychotherapy has the disadvantage of being expensive.^[6]^ In recent years, acupuncture has been recognized by most patients. Studies have shown that acupuncture is effective for depression, and it also has a therapeutic effect on the improvement of cardiac function in patients with HF.^[21,22]^ Therefore, this study is the first to explore the safety and efficacy of acupuncture for anxiety and depression in patients with HF through systematic review and meta-analysis. It aims to provide clinicians with valuable references to complementary therapy and alternative therapy.

## Author contributions

**Conceptualization:** Cheng Wang, Jia Wang.

**Data curation:** Cheng Wang, Mingpeng Shi,Miao Zhang.

**Formal analysis:** Jia Wang, Keying Yu, Ruozhu Lu.

**Funding acquisition:** Yue Deng.

**Investigation:** Miao Zhang.

**Methodology:** Cheng Wang, Jia Wang.

**Project administration:** Mingpeng Shi, Shi Rui.

**Resources:** Cheng Wang, Shi Rui, Keying Yu, Ruozhu Lu.

**Software:** Cheng Wang, Jia Wang.

**Supervision:** Yue Deng.

**Validation:** Cheng Wang, Yue Deng.

**Visualization:** Yue Deng.

**Writing – original draft:** Cheng Wang.

**Writing – review & editing:** Yue Deng.
